# Protein superfolds are characterised as frustration-free topologies: A case study of pure parallel *β*-sheet topologies

**DOI:** 10.1371/journal.pcbi.1012282

**Published:** 2024-08-07

**Authors:** Hiroto Murata, Kazuma Toko, George Chikenji

**Affiliations:** Department of Applied Physics, Nagoya University, Nagoya, Aichi, Japan; Harvard University, UNITED STATES OF AMERICA

## Abstract

A protein superfold is a type of protein fold that is observed in at least three distinct, non-homologous protein families. Structural classification studies have revealed a limited number of prevalent superfolds alongside several infrequent occurring folds, and in *α*/*β* type superfolds, the C-terminal *β*-strand tends to favor the edge of the *β*-sheet, while the N-terminal *β*-strand is often found in the middle. The reasons behind these observations, whether they are due to evolutionary sampling bias or physical interactions, remain unclear. This article offers a physics-based explanation for these observations, specifically for pure parallel *β*-sheet topologies. Our investigation is grounded in several established structural rules that are based on physical interactions. We have identified “frustration-free topologies” which are topologies that can satisfy all the rules simultaneously. In contrast, topologies that cannot are termed “frustrated topologies.” Our findings reveal that frustration-free topologies represent only a fraction of all theoretically possible patterns, these topologies strongly favor positioning the C-terminal *β*-strand at the edge of the *β*-sheet and the N-terminal *β*-strand in the middle, and there is significant overlap between frustration-free topologies and superfolds. We also used a lattice protein model to thoroughly investigate sequence-structure relationships. Our results show that frustration-free structures are highly designable, while frustrated structures are poorly designable. These findings suggest that superfolds are highly designable due to their lack of frustration, and the preference for positioning C-terminal *β*-strands at the edge of the *β*-sheet is a direct result of frustration-free topologies. These insights not only enhance our understanding of sequence-structure relationships but also have significant implications for de novo protein design.

## Author summary

A protein superfold is a protein fold that appears in at least three different non-homologous protein families. Superfolds are unique in their ability to accommodate multiple functions within a single fold, a feature not typically seen in other folds. Studies in structural classification have led to two notable observations: the existence of a limited number of common superfolds contrasted with a larger variety of less frequent folds, and a recurring pattern in *α*/*β* type superfolds where the C-terminal *β*-strand often occupies the edge of the *β*-sheet, while the N-terminal *β*-strand is usually found in the middle. The origins of these patterns, whether they stem from evolutionary sampling bias or physical interaction mechanisms, remain unclear. This article provides a physics-oriented explanation for these observations, specifically concentrating on pure parallel *β*-sheet topologies. The insights gained from this research are crucial in enhancing our understanding of the relationship between protein sequences and structures, and are expected to contribute significantly to the de novo design of new proteins.

## 1 Introduction

Research in protein structural classification has significantly advanced our understanding of the relationships between sequence, structure, function, and evolution [1–3]. These studies have also raised important new questions. One such question pertains to the relationship between protein folds and superfamilies. A protein fold is defined by the order and orientation of secondary structure elements, while a superfamily represents the largest grouping of proteins for which common ancestry can typically be inferred based on structural and functional similarity [4]. Orengo *et al*. reported that many protein folds are exclusive to a single superfamily; the majority of protein folds are unique to a specific superfamily [5]. Conversely, a very small number of distinct protein folds were found across multiple superfamilies. These distinctive protein folds, observed in three or more superfamilies, are termed superfolds [5]. Interestingly, despite the existence of only nine superfolds, as many as 30% of the domains cataloged in CATH are part of these superfolds. This observation was first documented in 1994, and this trend has continued through 2021 [6]. The reasons behind the popularity of these superfolds remain an enigma.

Several structural characteristics of superfolds have been proposed to date, including pronounced symmetry [7], a significant presence of super secondary structure [8], and a limited number of jumps in *β*-sheet proteins [9]. However, it has been observed that there are folds that meet all these criteria and are thus considered potential superfolds, but these folds are either not prevalent or do not exist in the database [10, 11]. One such category is the reverse fold of superfolds, which is obtained by reversing the N- to C-terminal chain direction of a given fold. Past studies have reported that the reverse fold of the superfold is either non-existent in the database or exists in small numbers [12]. Jane Richardson, in her well-known 1981 review, stated, *“There must be some strong reason why it is so rare”* [13]. However, the reason remains unknown to this day. This is another unresolved issue raised by structural classification studies. Closely related to the issue of chain reversal is an interesting observation of the *αβ* type fold: the C-terminal *β*-strand is often located at the edge of the *β*-sheet, while the N-terminal *β*-strand tends to be in the middle of the *β*-sheet in the protein structure database [12]. The reason why the C-terminal *β*-strand strongly prefers the edge of the *β*-sheet is also, as far as we know, still unresolved.

The aim of this paper is to offer a physics-based perspective to address these questions. We focus exclusively on parallel *β*-sheet topologies with 3–6 *β*-strands for our case study. Initially, we utilized a recent database to conduct a statistical analysis, confirming that (1) superfolds make up only a fraction of all theoretically possible patterns, (2) superfolds tend to position the C-terminal *β*-strand at the edge of *β*-sheets, and (3) reverse superfolds are rare or non-existent. Subsequently, we propose a simple theory to elucidate the differences in physical properties between superfolds and their reverse counterparts. This theory shows that superfolds can simultaneously satisfy multiple physical rules, while reverse superfolds cannot. In this paper, we categorize folds that can simultaneously satisfy the physical rules as frustration-free folds, and those that cannot as frustrated folds. The theory further illustrates that frustration-free folds represent only a fraction of all theoretically possible patterns and explains why the C-terminal *β*-strand has a strong preference for the edge of *β*-sheets in frustration-free topologies. Lastly, we conducted a comprehensive exploration of sequences and structures using a lattice protein model, demonstrating that frustration leads to low designability, while non-frustration results in high designability. These findings suggest that superfolds are those with high designability due to the absence of frustration.

## 2 Results

### 2.1 The database analysis

This research evaluated all theoretically possible parallel *β*-sheet topologies comprising 3 to 6 *β*-strands. We computed the Occurrence Frequency of Homologous-group in a Topology (OFHT) for each topology within a protein structure database. This analysis aimed to confirm high skewness in the distribution of protein folds and the infrequency of reverse folds of superfolds. Here, OFHT is defined as the number of Homology-groups in a given topology (see the Materials and Methods section for details). This quantity is an indicator of how many diverse sequences have a given topology as their native structure. We employed the recent version of a semi-manually curated database, ECOD (version 20210511: develop280), which hierarchically classifies protein domains according to homology, reflecting their evolutionary relationship [14]. Utilizing the STRIDE program [15], we identified secondary structures and hydrogen bonds in the protein domains in this dataset. Following previous studies [9, 10, 12, 16], we defined *β*-sheet topologies in an abstract manner based on their *β*-strand connectivity and hydrogen bonding pattern, i.e., the number, order, and orientation of constituent *β*-strands in a *β*-sheet. These *β*-strands are sequentially numbered along the protein’s backbone, starting from the N-terminus. A topology is characterised by the *β*-strand’s sequential positions and directional orientation. Assuming that all *βαβ*-units are right-handed, there are *n*!/2 theoretically possible pure parallel *β*-sheet topologies consisting of *n* *β*-strands, so the total number of 3–6 parallel *β*-sheet topologies is 435. Out of these, 167 topologies exhibit no clash between crossing connections.

**Fig 1:**
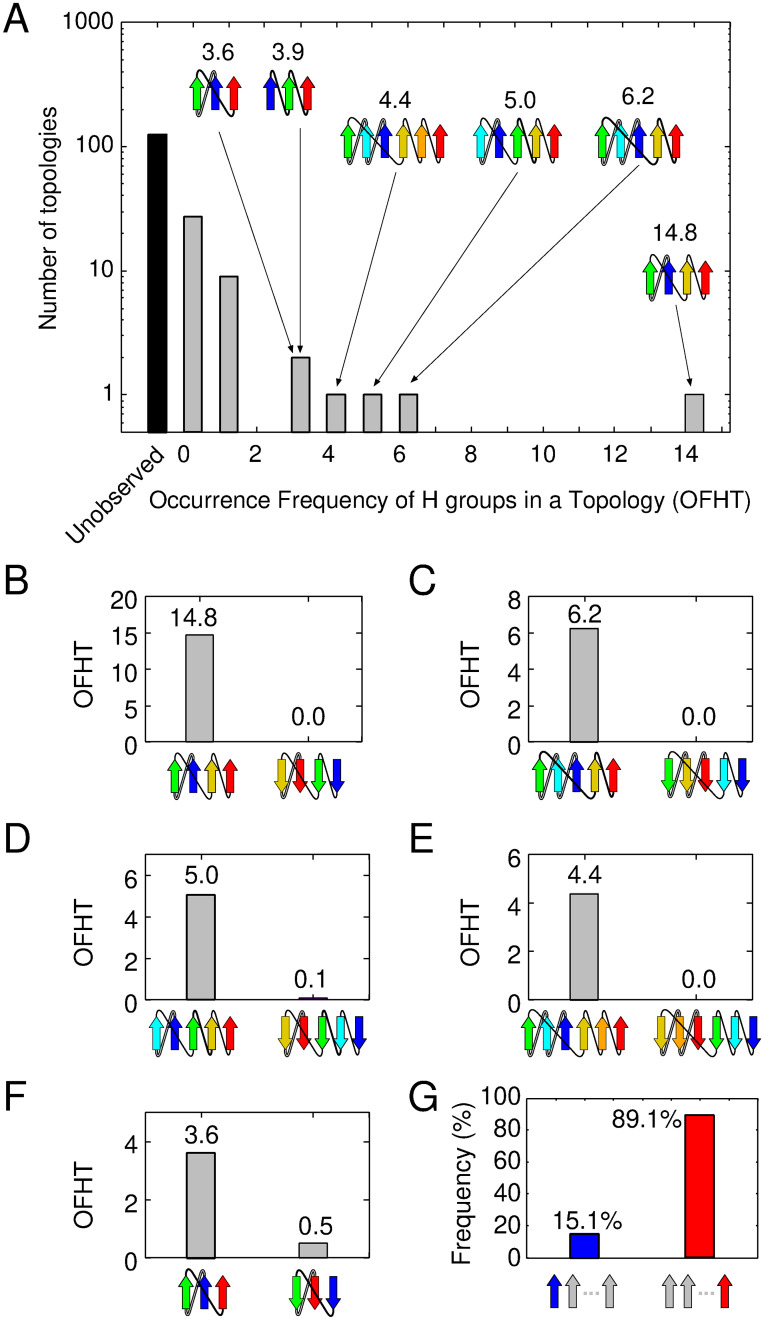
Occurrence frequency of homologous-group in a topology (OFHT) for each topology in the ECOD database. (A) It presents the distribution of pure parallel *β*-sheet topologies. The inset shows the topology diagrams of superfolds. Gray and black bars represent observed and unobserved topologies, respectively. (B)-(F) These panels depict the occurrence frequency of a superfold (on the left) and its reverse topology (on the right). (G) This panel calculates the probabilities of the N- and C-terminal *β*-strand being positioned at the edge of the *β*-sheet for all observed topologies.

This study involves an analysis of the OFHTs in 167 clash-free topologies. The analysis reveals a highly skewed distribution, aligning with previous research [5]. In Fig 1A, the vertical axis represents the number of topologies with a given OFHT (indicated by the horizontal axis). The distribution underscores a limited number of frequently occurring topologies alongside a larger quantity of less common ones. Following the terminology of Ref. [5], topologies with three or more OFHTs are termed “superfolds.” Six superfolds were identified in descending order of OFHT values: 2_↑_1_↑_3_↑_4_↑_, 3_↑_2_↑_1_↑_4_↑_5_↑_, 2_↑_1_↑_3_↑_4_↑_5_↑_, 3_↑_2_↑_1_↑_4_↑_5_↑_6_↑_, 1_↑_2_↑_3_↑_ and 2_↑_1_↑_3_↑_, as depicted in the inset of Fig 1A. In addition to the 6 superfolds, the dataset included 36 normal folds (defined as topologies with OFHT values between 0 and 3) and the remaining 125 topologies were not observed. It is noteworthy that these findings are based solely on pure parallel *β*-sheet proteins but are consistent with previous observations encompassing all classes of protein folds [5]. We examined whether the previous assertion regarding the rarity of reverse folds of superfolds remains valid within the current database. Our analysis of six superfolds in this dataset has revealed five topologies that transform into a different topology when the direction of the N- to C-terminal chain is reversed. The only exception is 1_↑_2_↑_3_↑_, which retains its topology when reversed; hence, we exclude it from further consideration. We present a comparison of the OFHTs for these five pairs of superfolds and their corresponding reversals in Fig 1B–1F. In all of these pairs, reverse folds occur infrequently; they are either entirely absent or, at most, represent less than 1/7 of the corresponding superfold, aligning with prior findings [12]. Furthermore, as depicted in Fig 1B–1F, the C-terminal *β*-strand of the superfold consistently resides at the edge of the *β*-sheet, while the N-terminal *β*-strand tends to occupy the middle of the *β*-sheet, in line with previous research [12]. We calculated the probabilities of the N- or C-terminal *β*-strand being positioned at the edge of the *β*-sheet for all observed topologies in this dataset, yielding percentages of 15.1% for the N-terminal *β*-strand and 89.1% for the C-terminal *β*-strand (Fig 1G). In contrast, assuming that 167 clash-free topologies occur with equal likelihood, both the N- and C-terminal *β*-strands are located at the end of the *β*-sheet with a probability of 37.1%. These values differ significantly from the results of the database analysis, suggesting a compelling rationale behind the strong preference of the C-terminal *β*-strand for the edge of the *β*-sheet.

### 2.2 The theory for explaining the differences in physical properties between superfolds and their reverse folds

This section introduces a theory that provides a physical explanation for why a superfold is more favorable than its reverse fold. The term “physically favorable” refers to a topology’s ability to simultaneously comply with three specific rules outlined below.

*Rule I. The right-handed rule for crossover connections of βαβ-unit*.

**Fig 2:**
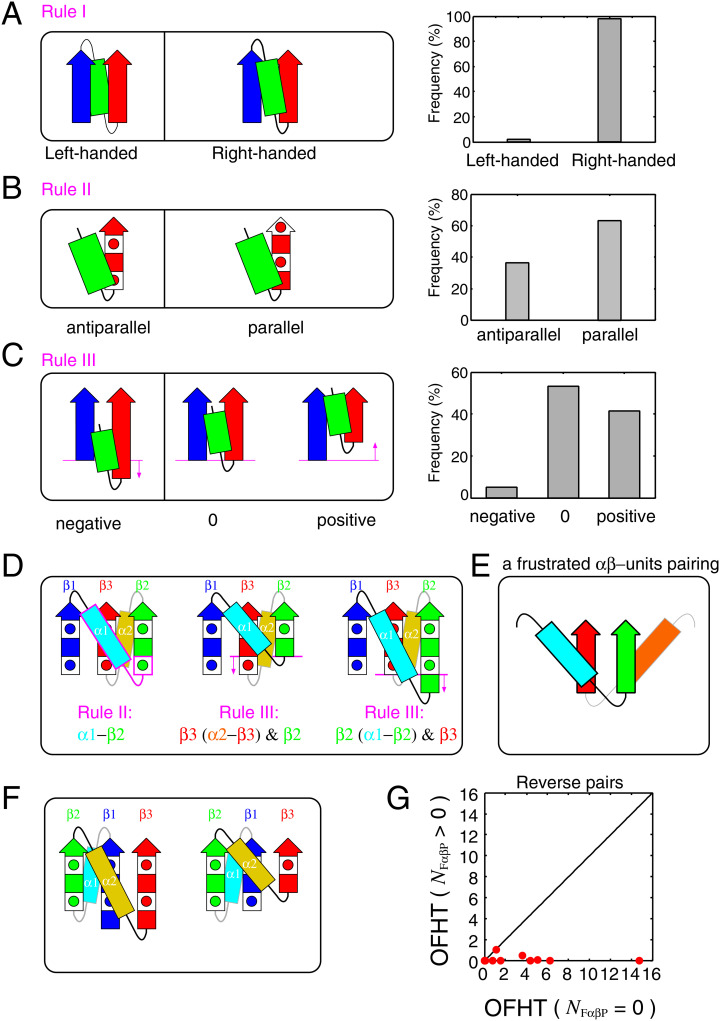
The basic rules and the frustrated *αβ*-units pairing. (A) The right-handed rule for crossover connections of *βαβ*-units is depicted. The left side shows schematics of left- and right-handed *βαβ*-units, while the right side presents their occurrence frequencies in the dataset. The arrows represent *β*-strands and the rectangles represent *α*-helices. (B) The *αβ*-rule is represented. The left side displays schematics of antiparallel and parallel *αβ*-units, and the right side shows their occurrence frequencies in the dataset. The square containing a red-filled circle symbolizes a single amino acid residue with a side chain located on the proximal side, and the red-colored filled square represents a residue with a side chain located on the opposite side. (C) The register shift rules for a *β*-strand of an *αβ*-unit and its neighboring *β*-strand are depicted. The left side provides a schematic representation of negative, zero, and positive register shifts, and the right side shows their occurrence frequencies in the dataset. (D) Schematics of structures with a topology that cannot simultaneously satisfy the three rules. The areas that violate the rules are highlighted in magenta. Beneath each schematic, a violated rule is documented. (E) A schematic of a frustrated *αβ*-units pairing is shown. (F) Schematics of structures with a topology that can simultaneously satisfy the three rules. (G) OFHTs of topology pairs are presented, where one is a topology that does not contain an F*αβ*P (the horizontal axis) and the other is its reverse topology that does (the vertical axis).

Crossover connections in the *βαβ*-unit predominantly exhibit right-handedness [17, 18]. This rule applies not only to the two connected *β*-strands that are nearest neighbors in the *β*-sheet but also to those *β*-strands that are connected yet separated by one or more intervening *β*-strands in the *β*-sheet [17]. The rationale for this rule lies in thermodynamic stability and kinetic accessibility [19–21]. Notably, over 98% of the *βαβ*-motifs in the database display right-handedness [21], a finding corroborated by our recent calculations (Fig 2A). For detailed methods on calculating the handedness of *βαβ*-units, please refer to Ref. [22].

*Rule II. The αβ-rule*.

The *αβ*-units exhibit a preference for a parallel orientation [23]. This means that the vector from the *α*-helix to the *β*-strand of an *αβ*-unit tends to align parallel to the C*α*-C*β* vector of the first residue in the strand. When the two vectors are antiparallel, the orientation of *αβ*-units becomes antiparallel (see Fig 2B). This rule, discovered through database analysis and confirmed in physics-based simulations, is believed to result from physical interactions [23]. This rule is not as strict as the right-handed rule of *βαβ*-units, with a ratio of approximately 65:35 for parallel to antiparallel orientations (see Fig 2B).

*Rule III. The N-terminal register shift rules for a β-strand of an αβ-unit and its neighboring β-strand*.

The register shift between the N-terminal residue of the *β*-strand of the *αβ*-unit and the N-terminal residue of its neighboring *β*-strand strongly disfavors negative values [24]. To clarify, the residue offset between the N-terminal residue of the *β*-strand of the *αβ*-unit and the N-terminal residue of the *β*-strand to its left when viewed from a specific direction, should not be negative [24]. This specific direction is defined as the one where the *α*-helix of the *αβ*-unit is closer to the *β*-strand and the *β*-strand is facing upwards (Fig 2C). The sign of the register shift is defined as positive when the N-terminal residue of the *β*-strand of the *αβ*-unit is located above the N-terminal residue of the *β*-strand to its left, and negative when the opposite is the case (see the left side of Fig 2C). For a detailed and comprehensive definition, please refer to Ref. [24]. In this context, “negative register shifts” are strongly discouraged, as indicated by database analysis (see the right side of Fig 2C); they were observed for only about 5% of the entire data set. The physical mechanism that prevents negative register shifts has been clarified through all-atom model calculations and exhaustive structure sampling [24]. It is important to note that the blue *β*-strand and the green *α*-helix shown in Fig 2C do not need to be directly connected [24].

In this section, we clarify how certain topologies can simultaneously satisfy the three aforementioned rules, while others cannot. We use 2_↑_1_↑_3_↑_, depicted on the left side of Fig 1F, as an example of a topology that can comply with all the three rules, and 1_↑_3_↑_2_↑_ (equivalent to 2_↓_3_↓_1_↓_), also shown on the right side of Fig 1F, as an example of a topology that cannot. It is important to note that these two topologies are reverses of each other. In the ensuing discussion, we will assume that the right-handed rule of the *βαβ*-motif always holds. We will refer to the *β*-strands in these topologies as *β*1, *β*2, and *β*3, starting from the N-terminal side. The *α*-helix connecting *β*1 and *β*2 will be referred to as *α*1, and the one connecting *β*2 and *β*3 as *α*2 (see Fig 2D and 2F). Firstly, we will explain why 1_↑_3_↑_2_↑_ cannot simultaneously adhere to all the three rules in three steps.

**1st step**: When the N-terminal residues of *β*2 and *β*3 are positioned at the same height as shown on the left side of Fig 2D, neither the *αβ*-unit consisting of *α*1 and *β*2 nor the one consisting of *α*2 and *β*3 can satisfy Rule II. As illustrated on the left side of Fig 2D, the *αβ*-unit consisting of *α*1 (cyan) and *β*2 (green) violates Rule II, as the vector from the *α*-helix to the *β*-strand is antiparallel to the C*α*-C*β* vector of the first residue of *β*2 (the region breaking the rule is outlined in magenta). Conversely, if the N-terminal side chains of *β*2 and *β*3 are on the other side of the paper, the *αβ*-units consisting of *α*2 (yellow) and *β*3 (red) violate Rule II. Therefore, when the register shift of the N-terminal side of *β*2 and *β*3 is zero, Rule II cannot be satisfied, regardless of the orientation of the side chains of the N-terminal residues of *β*2 and *β*3.

**2nd step**: If the N-terminal residue of *β*2 is located above the N-terminal residue of *β*3, Rule III cannot be satisfied because the N-terminal register shift between *β*2 and the *β*-strand of the *αβ*-unit consisting of *α*2 and *β*3 is negative (see the middle of Fig 2D).

**3rd step**: If the N-terminal residue of *β*2 is located below the N-terminal residue of *β*3, Rule III cannot be satisfied because the N-terminal register shift between *β*3 and the *β*-strand of the *αβ*-unit consisting of *α*1 and *β*2 is negative (see the right side of Fig 2D).

Considering these three steps, the 1_↑_3_↑_2_↑_ topology cannot simultaneously satisfy all the three rules for any offset value between the N-terminal residues of *β*2 and *β*3. Broadening the scope of the discussion, a topology cannot meet all the three rules if it contains two *αβ*-units that form parallel *β*-sheets, with each *α*-helix located on opposite sides of the *β*-sheet, as depicted in Fig 2E. We refer to such a pair of *αβ*-units as a frustrated *αβ*-units pairing (F*αβ*P) and denote the number of F*αβ*Ps in a topology as *N*_F*αβ*P_. Note that a reverse structure of an F*αβ*P is not frustrated. A detailed explanation of this fact is provided in the Materials and Methods section.

Next, we explain how the 2_↑_1_↑_3_↑_ topology can simultaneously fulfill all the three rules. This topology does not involve F*αβ*P, indicating that it can satisfy all the three rules simultaneously. In fact, there are certain arrangements of *β*-strands that can meet all the three rules. Fig 2F provides two examples of such *β*-strand arrangements for the 2_↑_1_↑_3_↑_ topology.

The observation that a superfold lacks F*αβ*P, while its reversal with infrequent occurrences contains it, holds true for all pairs of superfolds and their reversals, not just the specific pair of 1_↑_3_↑_2_↑_ and 2_↑_1_↑_3_↑_ topologies. It can be readily confirmed that the superfolds presented in Fig 1B–1F also lack F*αβ*P, while their reversals contain it. This observation suggests that the absence of F*αβ*P is one of the necessary conditions for a topology to be classified as a superfold. This implication is further supported by the distribution data obtained from topologies with *N*_F*αβ*P_ = 0 and those with *N*_F*αβ*P_ > 0; all superfolds are found within the category of topologies with *N*_F*αβ*P_ = 0, whereas topologies with *N*_F*αβ*P_ > 0 are not designated as superfolds (S1A and S1B Fig). However, it is important to emphasize that having *N*_F*αβ*P_ = 0 alone does not suffice as a condition for a topology to be recognized as a superfold.

The condition *N*_F*αβ*P_ = 0 is not the only criterion for classifying a topology as a superfold; there are other factors to consider. One such factor is the small number of jumps (*N*_j_) (S1C and S1D Fig). *N*_j_ is defined as the count of pairs of *β*-strands that are adjacent in sequence but not neighboring in the *β*-sheet structure. Prior studies have emphasized that topologies with more than one jump (*N*_j_ > 1) are strongly discouraged within the Protein Data Bank (PDB) [9]. This discouragement stems from the fact that topologies with a high number of jumps encounter significant challenges during the folding process, primarily due to the substantial free energy barrier resulting from rapid entropy loss [25]. Therefore, both the number of frustrated *αβ*-units pairings and the number of jumps are key determinants in the classification of a topology as a superfold.

However, it is important to emphasize that only the former factor, the number of F*αβ*Ps, can provide insights into the differences in physical properties between pairs with reversed chain orientations, potentially influencing the OFHTs, as illustrated in Fig 1B–1F. This relationship extends beyond these reversed pairs and applies to all such pairs. Among all theoretically possible topologies, there are 23 topology pairs where one lacks F*αβ*P, while its reverse contains F*αβ*P. Fig 2G presents a graphical representation of the OFHTs for these 23 topology pairs. In this plot, each pair’s OFHT with *N*_F*αβ*P_ = 0 is plotted on the horizontal axis, while the OFHT of its reverse topology with *N*_F*αβ*P_ > 0 is depicted on the vertical axis. Notably, all data points on the plot are located exclusively on the lower right-hand side of the diagonal line. This observation underscores the significant impact of the presence of F*αβ*P on the reduction of OFHTs.

### 2.3 Superfolds are characterised as frustration-free topologies

**Fig 3:**
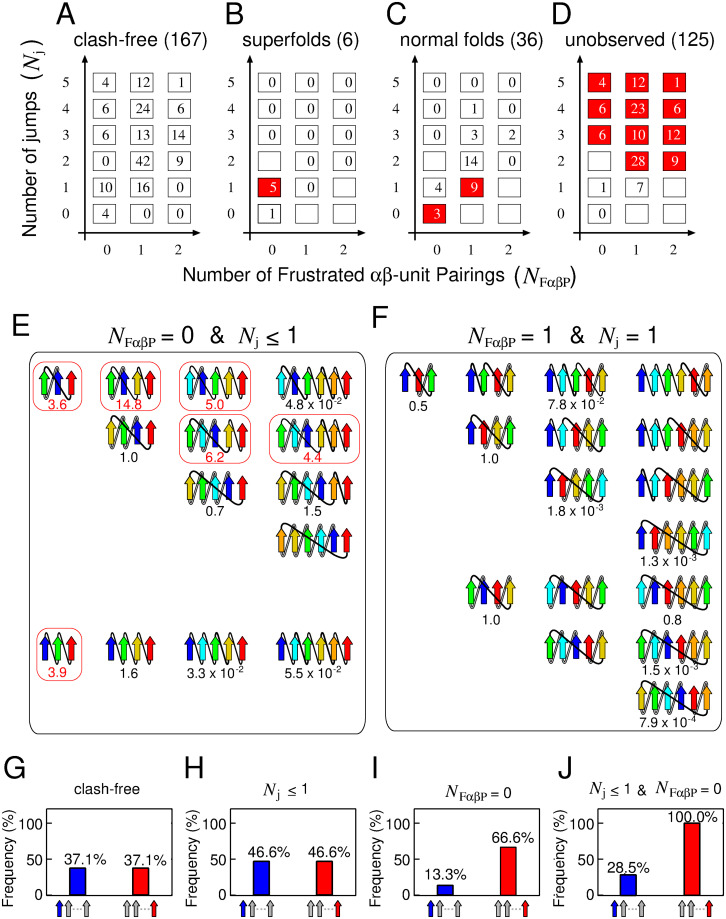
Topologies categorized by *N*_F*αβ*P_ and *N*_j_. (A)-(D): The number of topologies for theoretically possible clash-free states, superfolds, normal folds, and unobserved folds, depicted on a two-dimensional grid. The states highlighted in red indicate instances where more than 50% of the total number of theoretically possible clash-free topologies are observed. (E)-(F): Topology diagrams in the states (*N*_F*αβ*P_ = 0, *N*_*j*_ ≤ 1) and (*N*_F*αβ*P_ = 1, *N*_*j*_ = 1). The numbers below the topology diagrams represent their OFHTs. Topology diagrams surrounded by a red box represent superfolds. (G)-(J): The probabilities of the N- or C-terminal *β*-strand being at the edge of the *β*-sheet calculated from clash-free topologies, topologies with (*N*_j_ ≤ 1), those with (*N*_F*αβ*P_ = 0), and those with (*N*_j_ ≤ 1, *N*_F*αβ*P_ = 0).

In this section, we undertake a more comprehensive characterization of superfolds, focusing on two pivotal factors: the number of F*αβ*Ps and the number of jumps within a *β*-sheet. Fig 3A–3D depict the distribution of topologies across four distinct categories: theoretically possible clash-free topologies, superfolds, normal folds, and unobserved folds. These topologies are charted on a 3 × 6 two-dimensional grid, where the horizontal axis signifies the number of F*αβ*Ps (*N*_F*αβ*P_), and the vertical axis represents the number of jumps (*N*_j_). Notably, states highlighted in red denote instances where over 50% of the theoretically possible clash-free topologies within the states fall into the categories of superfolds (Fig 3B), normal folds (Fig 3C), or unobserved folds (Fig 3D). The initial salient observation is that the number of topologies meeting both physically preferred conditions, *N*_F*αβ*P_ = 0 and *N*_j_ ≤ 1, is notably lower than the total count (Fig 3A). This implies a constrained number of topologies adhering to physically favorable criteria. The second critical insight is that all six superfolds are concentrated within states characterised by *N*_F*αβ*P_ = 0 and *N*_j_ ≤ 1 (Fig 3B). Conversely, topologies falling within grids defined by *N*_F*αβ*P_ = 0 and *N*_j_ ≤ 1 are likely to be superfolds, with 6 out of 14 topologies being superfolds. Remarkably, within the grid where (*N*_F*αβ*P_ = 0, *N*_j_ = 1), as many as 50% (5 out of 10) of topologies are superfolds. These observations suggest that superfolds can be effectively characterised as topologies that satisfy all physically favorable requirements (*N*_F*αβ*P_ = 0, *N*_j_ ≤ 1). Henceforth, we will denote a topology meeting all these criteria (*N*_F*αβ*P_ = 0, *N*_j_ ≤ 1) as a frustration-free topology, while one that fails to meet these criteria will be termed a frustrated topology. The third crucial point is that as topologies deviate further from the state of (*N*_F*αβ*P_ = 0, *N*_j_ = 1), there is a discernible decrease in the overall OFHT (Fig 3C and 3D). This implies that both of these variables contribute significantly to distinguishing between superfolds, normal folds, and unobserved folds. Based on these findings, we propose the “frustration-free hypothesis of superfolds”: a superfold is a topology that impeccably satisfies all various physical rules. Subsequently in this paper, we will present data to support this hypothesis from a lattice protein model study.

Next, we delve into the characteristics of topologies within different grid states. We specifically present the topologies in two states: (*N*_F*αβ*P_ = 0, *N*_*j*_ ≤ 1) and (*N*_F*αβ*P_ = 1, *N*_*j*_ = 1). The topologies of the other grids are detailed in S2–S7 Figs.

The first example pertains to the state with (*N*_F*αβ*P_ = 0, *N*_*j*_ ≤ 1). The state consists of 14 clash-free topologies (Fig 3E). Out of these, six topologies display superfolds, while the other eight do not. Some of the eight non-superfolds can be interpreted as physically unfavorable using known facts other than the two features used here. The first such class is topologies in which local contacts are dominant among all contacts. One potential explanation for this class not being superfolds is their small number of non-local contacts, leading to low cooperativity as anticipated [26, 27]. This characteristic might be a factor making them less likely to be superfolds. We examined the relationship between the locality of the topology and its occurrence frequency in the database for all 167 clash-free topologies. As a quantitative measure of locality of the topology, we introduced the *β*-sheet-topology Relative Contact Order (*β*RCO), which is a variant of the Relative Contact Order (RCO), a measure of the locality of the inter-amino acid contacts in the native structure [28]. Refer to the Materials and Methods section for the definition of the *β*RCO and the RCO. Our findings revealed that all superfolds exhibited intermediate *β*RCO, while topologies with small (< 0.2) or large (>0.5) *β*RCO are not superfolds (S8A Fig). This observation suggests that having intermediate *β*RCO is one of the necessary conditions for being superfolds. Not meeting this necessary condition may be the reason why the three topologies, 1_↑_2_↑_3_↑_4_↑_5_↑_6_↑_, 1_↑_2_↑_3_↑_4_↑_5_↑_, and 2_↑_1_↑_3_↑_4_↑_5_↑_6_↑_, are not superfolds. The second class is topologies with crossover connections that skip more than two intervening *β*-strands in the sheet. It includes 4_↑_3_↑_2_↑_1_↑_5_↑_, 4_↑_3_↑_2_↑_1_↑_4_↑_5_↑_, and 5_↑_4_↑_3_↑_2_↑_1_↑_6_↑_ topologies (S8B Fig). We refer to this type of crossover connection as a long-distance crossover connection. One possible explanation for this class not being superfolds is that the presence of long-distance crossover connections lead to high free energy barriers during the folding due to rapid entropy losses [25] and it is anticipated to be physically unfavorable, thereby disqualifying it as a superfold. As for the remaining two topologies, 1_↑_2_↑_3_↑_4_↑_ and 3_↑_2_↑_1_↑_4_↑_, it is currently unclear why these are not superfolds (S8B Fig).

The second example pertains to the state with (*N*_F*αβ*P_ = 1, *N*_*j*_ = 1), which does not contain a superfold and has high probability of a normal fold (9/16). This state comprises reverse topologies of (*N*_F*αβ*P_ = 0, *N*_*j*_ = 1) (upper 4 rows) and other topologies (lower 3 rows) (Fig 3F). The reverse topologies depicted in the lower 3 rows correspond to either identical to the original topology or another one within this category. Notably, the difference in the physical properties of topologies with (*N*_F*αβ*P_ = 0, *N*_*j*_ ≤ 1) and those with (*N*_F*αβ*P_ = 1, *N*_*j*_ = 1) has previously gone unrecognized. However, F*αβ*P has revealed their distinct physical characteristics. Our hypothesis suggests that topologies within this state cannot achieve superfold status due to the presence of F*αβ*P.

In the final segment of our 2D plane analysis, we demonstrate that the likelihood of the N- or C-terminal *β*-strands being located at the edge of the *β*-sheet aligns closely with database observations (Fig 1G). This alignment is particularly noticeable when the topologies meet specific conditions that are physically favorable (*N*_j_ ≤ 1, *N*_F*αβ*P_ = 0). In the following discussion, we assume that all topologies occur with equal probability. Fig 3G provides a visual representation of the probability of an N- or C-terminal *β*-strand being located at the edge of the *β*-sheet across all 167 clash-free topologies. It becomes apparent that the N- and C-terminal *β*-strands have an equal probability (37.1%) of occupying the end of the *β*-sheet. Fig 3H presents the probabilities calculated for the 30 topologies that meet the *N*_j_ ≤ 1 condition. Here, the N- and C-terminal *β*-strands have an identical likelihood of being situated at the edge of the *β*-sheet. It is clear that the *N*_j_ ≤ 1 condition alone cannot make a difference to the probability of N- and C-terminal *β*-strands being at the edge of the *β*-sheet. When we perform similar calculations for the 30 topologies that meet the *N*_F*αβ*P_ = 0 condition (Fig 3I), we observe a difference in the probability distribution between the N- and C-terminal *β*-strands. The C-terminal *β*-strand has a higher likelihood of being located at the edge than the N-terminal *β*-strand. This finding underscores the significant impact of the presence or absence of F*αβ*P on the preferred positioning of the N- and C-terminal *β*-strands. Topologies that satisfy both conditions, *N*_F*αβ*P_ = 0 and *N*_j_ ≤ 1 (Fig 3J), show an even higher probability of the C-terminal *β*-strand being located at the edge. This observation aligns with the findings in Fig 1G. Collectively, these insights suggest that the prevalence of proteins with C-terminal *β*-strands at the edge of the *β*-sheet in the database can be attributed to the preference for frustration-free topology.

### 2.4 Frustration-free structures are highly designable: Implication from a lattice model study

In the previous section, we established a connection between superfolds and frustration-free topologies, demonstrating that all superfolds are frustration-free and that frustration-free topologies are often associated with superfolds. This leads us to the question: why are frustration-free protein topologies so common among various protein families? To address this, we utilize a modified version of the two-dimensional lattice HP model for proteins [29, 30], which incorporates unfavorable local interactions mimicking the rules illustrated in Fig 2A–2C.

For relatively short chains, the lattice HP model is especially suitable for examining sequence-structure relationships since it enables thorough exploration of both sequence and structure space [31]. Our investigation reveals that frustration-free structures exhibit high designability. Here, the statement that a structure is highly designable means that when all theoretically possible amino acid sequences are considered, substantial number of the sequences adopt the structure as their native state [31]. Given that a superfold is defined as a protein fold observed in a large number of non-homologous families, high designability is a prerequisite for a structure to be classified as a superfold.

**Fig 4:**
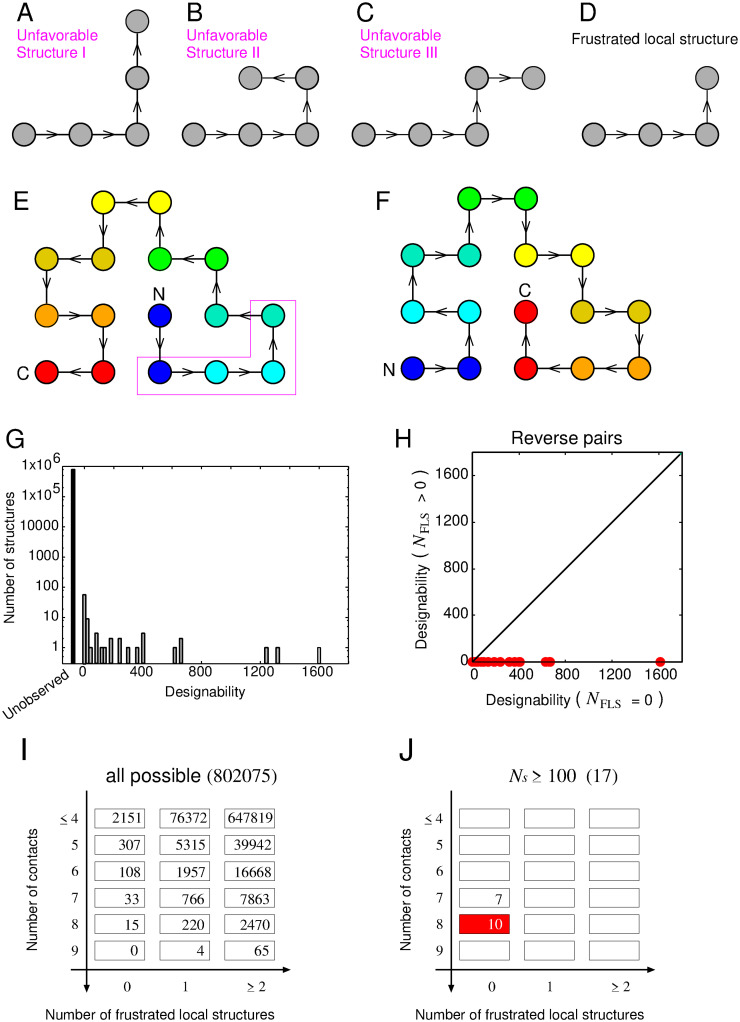
The lattice protein model employed in this study. (A)-(C) Depict three structures defined as physically unfavorable. Each filled gray circle symbolizes a single residue. Black lines with arrows depict covalent bonds; the arrows indicate the the N- to the C-terminal chain direction. (D) Represents a frustrated local structure (FLS), a local structure that necessarily contains one of the three physically unfavorable structures. (E) Provides an example of a structure containing an FLS. Rainbow coloring from blue to red indicates the N- to C-terminal position of the residues in the model. (F) Illustrates the reverse structure of (E), which does not contain an FLS. (G) Shows a histogram of designability for the lattice model with the local interactions. (H) Displays designabilities of structure pairs, where one is a structure that does not contain an FLS and the other is its reverse structure that does. (I)-(J) Illustrates the number of all possible structures and highly designable structures mapped onto the two-dimensional grid, *N*_F*αβ*P_ and *N*_FLS_.

In the lattice HP model, a protein conformation is represented as a self-avoiding path on a two-dimensional square lattice and an amino acid sequence is represented by a string using only two types of amino acids: hydrophobic (H) and polar (P). This simplistic representation facilitates a detailed exploration of the interplay between protein sequences and structures, especially for short protein chains. In the original HP model [29], the energy, denoted as *E*_HP_, associated with a protein chain’s conformation is solely determined by the number of hydrophobic–hydrophobic contacts, represented as *n*_HH_. This energy is expressed as *E*_HP_ = −*ϵ*_HH_ ⋅ *n*_HH_, where *ϵ*_HH_ is a positive constant. Beyond the original energy function, we introduce a sequence-independent energy penalty to emulate the effects shown in Fig 2A–2C. We define three specific local structures, as visualized in Fig 4A–4C, as inherently unfavorable structures. Each of these structures incurs a positive energy penalty, represented by *ϵ*_penalty_. The total energy penalty for a given structure is given by *E*_penalty_ = *ϵ*_penalty_ ⋅ *n*_penalty_, where *n*_penalty_ denotes the number of such unfavorable structures within a given configuration. Consequently, the overall energy, *E*, of a given protein structure is computed as *E* = *E*_HP_ + *E*_penalty_. Considering that the structure depicted in Fig 4D necessarily includes one of the three structures presented in Fig 4A–4C, and is therefore frustrated, we term this structure a ‘Frustrated Local Structure’ (FLS). An example of a structure containing an FLS is illustrated in Fig 4E. It should be mentioned that when this structure resides at the most C-terminal end, as shown in Fig 4F, no energetic penalty is imposed due to the absence of the structures illustrated in Fig 4A–4C. Importantly, the structure depicted in Fig 4F, the reverse of the Fig 4E structure, is devoid of frustration. Thus, an FLS and an F*αβ*P are analogous in that both are frustrated, while their reverse structures are not frustrated. A more detailed description of the model and the analogy is provided in the Materials and Methods section.

In this study, we conducted an analysis of protein chains composed of 16 monomers, resulting in a total of 802,075 conformations for this chain length. For each given sequence, we computed the energies of all structures using the parameters *ϵ*_HH_ = 1.0 and *ϵ*_penalty_ = 2.0. As will be shown later, the results are robust against the parameter value of *ϵ*_penalty_. Following previous studies, only sequences exhibiting a unique ground state are categorized as protein-like [31]. We exhaustively enumerated all possible conformations for the all possible 2^16^ sequences and identified those meeting the criteria for being protein-like, resulting in a total of 10,139 such sequences. Following this, we calculated the designability for each structure. In this context, the designability of a specific structure (*S*) is defined as the number of sequences (*N*_*S*_) that have the structure *S* as their unique ground state. It has been observed that in the HP model, there is a considerable variation in the designability of structures. A small number of structures are highly designable, while a significant number of structures exhibit low designability [31].

Our model, which incorporates local interactions, mirrors the findings of numerous previous studies on lattice protein models [31–35]. It exhibits a limited number of highly designable structures and a substantial number of structures with low designability (see Fig 4G). This observation aligns qualitatively with the data presented in Fig 1A, suggesting that the relationship between the number of folds and families within the database can be elucidated by examining the interplay between structure and sequence in our current model. To investigate the influence of FLSs on designability, we categorized all 802,075 structures into two distinct groups: those with FLSs and those without any FLS. We then plotted the relationship between designability and the number of structures for each group (see S9A and S9B Fig). Our investigation revealed a consistent pattern: all structures characterised by high designability were devoid of an FLS, while structures containing FLSs consistently exhibited low designability (S9A and S9B Fig). These findings suggest that the absence of an FLS is a prerequisite for a structure to exhibit high designability.

While the absence of an FLS is a key factor in determining high designability, it is not the only criterion. There are other contributing factors, such as the number of contacts (*N*_*c*_), which is another crucial determinant of high designability (S9C and S9D Fig). Therefore, both presence of FLSs and the number of contacts influence designability. However, the presence of FLSs is the only factor that can account for differences in the designability between pairs with reversed chain orientation. For example, consider the structure with the highest designability (*N*_*s*_ = 1614) depicted in Fig 4F, and its reverse structure (*N*_*s*_ = 3) shown in Fig 4E. Both structures have the same number of contacts, making it impossible to distinguish them based solely on contact numbers. In contrast, the number of FLSs can differentiate these two cases: the structure with the highest designability lacks an FLS, while its reverse structure contains FLSs. This relationship extends beyond this specific pair and remains consistent across all pairs (Fig 4H). These findings underscore the pivotal role of the number of FLSs as one of the most critical determinants of designability, offering unique insights compared to other established determinants.

Highly designable structures can be more effectively characterised using two specific features: the number of FLSs (*N*_FLS_) and the number of contacts (*N*_c_). Fig 4I and 4J visually represent the number of all possible structures and highly designable structures (defined as *N*_s_ ≥ 100), mapped onto a 3 × 6 two-dimensional grid based on *N*_FLS_ and *N*_c_. The red-shaded region within Fig 4J indicates the state where over 50% of all possible structures within the state are highly designable. A significant observation from Fig 4I is that the number of structures satisfying both criteria (*N*_c_ ≥ 7, *N*_FLS_ = 0) is substantially smaller than the total number, suggesting a limited number of physically favorable structures. Notably, all 17 highly designable structures (Fig 4J) fall within the grids defined by (*N*_c_ ≥ 7, *N*_FLS_ = 0). Conversely, structures located within grids meeting these criteria (*N*_c_ ≥ 7, *N*_FLS_ = 0) are likely to be highly designable, with 17 out of 48 structures meeting this classification. The grid with (*N*_c_ = 8 and *N*_FLS_ = 0) is particularly significant, where as many as 66% (10 out of 15) structures exhibit high designability. Thus, these two variables prove to be effective in characterizing highly designable structures.

In addition to these two quantities (*N*_FLS_ and *N*_c_), there can be other quantities that characterise highly designable structures. For example, just as *β*RCO was a quantity characterizing superfolds in the database, RCO may be a quantity characterizing highly designable structures in the lattice model. To validate this, we investigated the relationship between RCO and designability for all possible 802,075 structures (S10 Fig). The results are consistent with those obtained from the database analysis, revealing that highly designable structures are associated with intermediate RCO. Conversely, structures exhibiting RCO less than 0.3 or exceeding 0.5 demonstrated poorly designable. This observation suggests that having intermediate RCO is one of the necessary conditions for being highly designable structures.

Results of the lattice protein model study shown above were obtained using the parameter *ϵ*_penalty_ = 2.0 for the energy calculation. We conducted calculations using different *ϵ*_penalty_ values (0.1, 0.5, 1.0, 3.0, 4.0) and confirmed the qualitative robustness of the results regardless of *ϵ*_penalty_’s value (see S11–S15 Figs). Specifically, the following four observations were qualitatively the same regardless the *ϵ*_penalty_ value: (i) The distribution of designabilities demonstrates a small proportion of highly designable structures and a significant number of poorly designable structures. (ii) Structures with smaller *N*_FLS_ and larger *N*_c_ generally have higher designability. (iii) Highly designable structures had intermediate RCO, and those with small or large RCO were poorly designable. (iv) Structures without any FLS have greater designabilities than their reverse counterparts that contain FLSs in most cases. Importantly, this last observation held true even when the *ϵ*_penalty_ was as small as 0.1, as shown in S11B Fig.

Applying the results derived from lattice model calculations to the database analysis of pure parallel *β*-sheet topologies, we can conclude that: (i) the frustration-free topologies exhibit high designability, thereby fulfilling the necessary condition for being superfolds, and (ii) the frustrated topologies cannot be designated as superfolds due to their low designability. It is crucial to underscore that the feature *N*_F*αβ*P_, which accounts for different physical properties linked to the reversal of chain direction, plays a pivotal role in addressing the two critical questions: why is the population of superfolds limited, and why do the reverse folds of the superfolds either not exist in the database at all or exist only in small numbers?

## 3 Discussions

What sets superfolds apart from other protein structures? Is their prevalence merely a result of evolutionary sampling bias, or is there a fundamental reason underlying their ubiquity? In this study, we provide compelling evidence that unequivocally sets superfolds apart as unique entities. Superfolds are characterised by their nature as frustration-free topologies, a feature that is relatively rare when compared to the vast array of all possible topologies. Notably, the identification of a frustration-free topology does not necessitate sequence information or energy calculations; it can be determined solely based on the topology and compliance with several physical rules. Our findings, derived from calculations using the HP lattice model, reveal that frustration-free structures exhibit significantly high designability. Therefore, we conclude that the widespread occurrence of superfolds across diverse protein families is primarily due to their elevated designability, rather than being merely a consequence of evolutionary sampling bias.

This study introduces the concept of frustration-free topology. The term ‘frustration’ was used in earlier studies, but it has a different meaning in this study. Here, we delineate these distinctions. Traditionally, ‘local frustration’ was defined through the changes in free energy that occur minor modifications in a protein’s sequence or structure [36]. A protein segment is typically deemed less frustrated if these perturbations result in a substantial increase in free energy. In contrast, minimal changes or reductions in free energy characterise a frustrated state. Previous investigations have demonstrated that these local frustrations are instrumental in identifying functional sites [37] and specific protein interactions [38], thereby facilitating protein functionality. Unlike previous studies, the methodology proposed in this study avoids using energy calculations and amino acid information and instead depends on a set of predefined structural rules. Therefore, the uniqueness of this method lies in its ability to define frustration purely based on topology.

**Fig 5:**
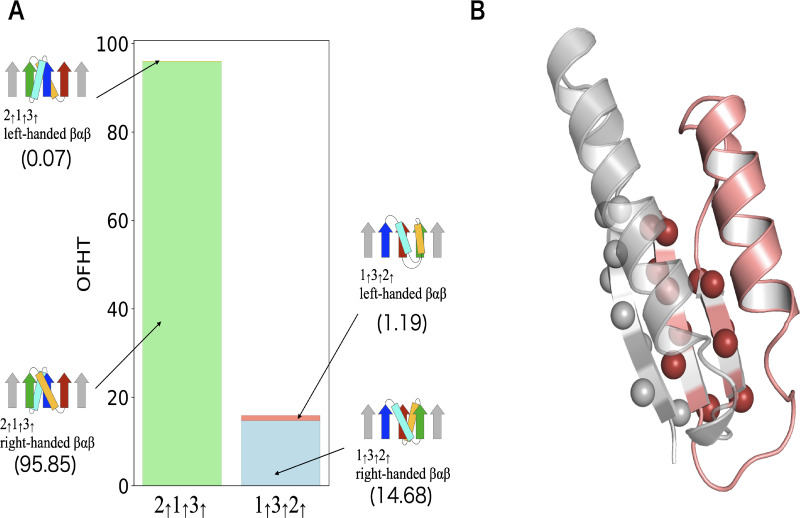
Left-handed *βαβ*-units in the dataset. (A) Depicts the occurrence frequencies of the left-handed and right-handed *βαβ*-units in the 2_↑_1_↑_3_↑_ and 1_↑_3_↑_2_↑_ substructures. The OFHT of topologies containing the 2_↑_1_↑_3_↑_ substructures is 95.92 among which the OFHT of topologies having the left-handed *βαβ*-unit is 0.07. The OFHT of topologies containing the 1_↑_3_↑_2_↑_ substructures is 15.87 among which the OFHT of topologies having the left-handed *βαβ*-unit is 1.19. (B) Provides an example of the structure containing the left-handed *βαβ*-unit. Displayed here is the 78–159 residues of chain A of the prenyl diphosphate synthase, Rv1086 (PDB ID: 2VG1). The left-handed *βαβ*-unit is colored in red. C*β* atoms of the *β*-sheet region are depicted as spheres.

Among the various principles that govern protein structures, Rule I, the right-handed rule for crossover connections of *βαβ*-units, is perhaps the most well-known. This rule states that the majority of *βαβ*-units are predominantly right-handed [17, 18]. However, in our dataset, we have identified a small subset of left-handed *βαβ*-units, as illustrated in Fig 2A. This raises the question: are there specific topological features where left-handed *βαβ*-units are frequently observed? To investigate this, we calculated the OFHTs for domains composed of four or more *β*-sheets containing the substructure 1_↑_3_↑_2_↑_ or 2_↑_1_↑_3_↑_. For each substructure, we computed the percentage of left-handed *βαβ*-units included in those substructures (see Fig 5A). Interestingly, the probability of encountering a left-handed *βαβ*-unit in domains containing 1_↑_3_↑_2_↑_ as a substructure (7.5%) is approximately 100 times higher than that in domains containing 2_↑_1_↑_3_↑_ (0.073%). This observation suggests the existence of a mechanism that favors the occurrence of left-handed *βαβ*-units in topologies containing 1_↑_3_↑_2_↑_. A plausible explanation for this mechanism is that domains containing 1_↑_3_↑_2_↑_ often prioritize satisfying Rule II and Rule III over Rule I, consequently leading to the adoption of left-handed *βαβ*-units. An example of such a structure that adheres to other rules while violating Rule I is depicted in Fig 5B. These findings suggest that in frustrated topologies, Rule I is often violated due to competition with other rules, making left-handed *βαβ*-units more likely to occur compared to frustration-free topologies.

Contrary to Rule I, the least stringent rule in defining an F*αβ*P is Rule II, as demonstrated in Fig 2. If Rule II were more stringent, like the other two rules, the observed topological diversity within the database would likely not be present. This conjecture stems from the outcome of the lattice model calculations, explained below. The lattice model calculation introduced three physically unfavorable local structures and applied an *ϵ*_penalty_ = 2 penalty to the energy for each unfavorable local structure. In this calculation, similar to the database analysis (see S1B Fig), many frustrated structures were observed as native structures (see S9B Fig). Yet, when the penalty was increased to *ϵ*_penalty_ = 4, these frustrated structures no longer appeared as native structures (see S15D Fig). This indicates that overly strict rules prevent the frustrated topologies, reducing diversity of protein topologies. An example of a protein with frustrated topology violating Rule II is adenylate kinase, a phosphotransferase enzyme that catalyzes the interconversion of the various adenosine phosphates (see S16 Fig). Such as this example, proteins with frustrated topology serve biologically important functions. Therefore, Rule II being less strict may be beneficial for the existence of these important proteins.

In this study, we have shown that it is possible to ascertain the presence or absence of frustration in a given protein topology by considering a combination of various structural rules. Moreover, we have found that frustrated topologies exhibit low designability. This discovery has significant implications for de novo protein design, as it enables us to estimate the rough designability of a given topology from topological information. The first example of this theory’s usefulness is its capacity to elucidate the factors contributing to the low success rates observed in certain de novo protein designs. In a study conducted by Rocklin *et al*. [39], where more than 1,500 proteins were de novo designed with four target topologies (*ααα*, *βαββ*, *αββα*, and *ββαββ*). It was observed that the *αββα* topology presented a substantially lower success rate compared to the others. Specifically, the success rate was only 2%, while the success rates for the remaining topologies ranged from 40% to 90%. This significant difference in the success rates of designing different target topologies raises an important question: what factors contribute to these disparities? We have previously reported that the *αββα* topology is a frustrated topology [22]. Given that frustrated topologies inherently exhibit lower designability, the limited success rate in designing the *αββα* topology can be attributed to its inherent low designability. The second example of this theory’s usefulness is its ability to predict protein topologies that are physically designable but are not found in nature. Fig 3C shows that weakly frustrated topologies, which are characterised by having low *N*_F*αβ*P_ and low *N*_j_, tend to be normal folds. This observation indicates that while weakly frustrated topologies are not as designable as superfolds, they are still likely to be designable. This indication is consistent with previous successes in de novo design of proteins with novel folds; indeed, all these novel folds are weakly frustrated topologies. For example, the Top7 topology (PDB ID: 1QYS) [40], known as the first successful de novo design of protein with a novel fold, satisfies all the rules presented in this paper and all other known rules [22], except for having *N*_j_ = 2. Another example of new fold protein design is the protein with a knot fold (PDB ID: 7BQD) [16], characterised by a topology of (N_F*αβ*P_ = 0, *N*_j_ = 3). In addition, although not classified as a novel fold, there have been documented successes in de novo design of proteins with p-loop fold (PDB ID: 2LVB) [23], characterised by a topology of (N_F*αβ*P_ = 1, *N*_j_ = 2). Based on the above discussion, it is postulated that proteins with a weakly frustrated novel topology (such as those listed in Fig 3F) are designable because these proteins are expected to exhibit designability on par with naturally occurring normal folds or achieve success rates of de novo design comparable to those documented in Ref. [16]. Thus, our theoretical framework for predicting the designability of a given topology will play a crucial role in exploring the protein fold space beyond what has been sampled through natural evolution.

## 4 Materials and methods

### 4.1 The datasets

To calculate the occurrence frequency of Homologous-groups in a topology, we utilized the ECOD database (date; 05/11/2021) [14], which contains 64,881 domains with sequence identity < 99%. The ECOD database classifies homologous protein domains according to categories of family and homology. The family (F) group comprises evolutionarily related protein domains with substantial sequence similarity, and the homology (H) group includes multiple F-groups with functional and structural similarities. The H-group corresponds to the superfamily in the other structural databases, such as SCOP [4] and CATH [1].

To identify the key rules for pure parallel *β*-sheet, as shown in Fig 2, we employed the PISCES server [41] with the criteria of sequence identity ≤ 25%, resolution ≤ 2.5Å and R-factor ≤ 0.3. We used *αβ*- and *βα*-units that meet specific criteria: the loop between the *α*-helix and the *β*-strand is shorter than ten residues, and the angle between them is less than 60 degrees, following Ref. [23].

### 4.2 Analysis of *β*-sheet topologies in the dataset

In the analysis based on secondary structure assignment using the STRIDE program [15], we selectively extracted pure parallel *β*-sheets adhering to specific criteria: (i) ensuring that all adjacent pairs of *β*-strands within the *β*-sheet are parallel, (ii) confirming that the *β*-sheet is open (not a barrel), (iii) verifying the absence of other *β*-sheets among the *β*-strands, and (iv) limiting the number of residues connecting two continuous sequential *β*-strands to fewer than 100. The process of deducing the topology from the results of secondary structure assignments followed the methodology outlined in Ref. [16].

### 4.3 Definition of the Occurrence Frequency of Homologous-group in a Topology (OFHT)

Using the ECOD dataset, we calculated the occurrence frequency of H-group in a topology *OFHT*(*T*) of a given topology *T* by summing the occupation ratio *OR*(*T*, *i*) of protein domains that have *T* in the *i*th H-group as

$$\begin{array}{c} OFHT\left(T\right)=\sum_{i=1}^{N_{\text{homology}}}OR\left(T,i\right), \end{array}$$

where *N*_homology_ is the total number of H-groups, and

$$\begin{array}{c} OR\left(T,i\right)=\frac{1}{N_{\text{family}}\left(i\right)}\sum_{j=1}^{N_{\text{family}}\left(i\right)}\frac{N_{\text{domain}}\left(T,i,j\right)}{N_{\text{domain}}\left(i,j\right)}. \end{array}$$

In this context, *N*_domain_(*T*, *i*, *j*) represents the number of protein domains with topology *T* in the *j*th F-group, which is part of the *i*th H-group in the dataset. *N*_domain_(*i*, *j*) signifies the total count of protein domains in the *j*th F-group, and *N*_family_(*i*) denotes the number of F-groups in the *i*th H-group.

### 4.4 Definition of the *β*-sheet-topology Relative Contact Order (*β*RCO)

The Relative Contact Order (RCO) of a protein is a measure of the locality of the inter-amino acid contacts in the native structure [28]. It is defined as the average sequence separation between all pairs of contacting residues normalized by the total sequence length:

$$\begin{array}{c} RCO=\frac{1}{L\cdot N}\sum^{N}\Delta S_{i,j}, \end{array}$$

where *N* is the total number of contacts, Δ*S*_*i*,*j*_ is the sequence separation between contacting residues *i* and *j*, and *L* is the total number of residues in the protein. Although this quantity is very useful, it cannot be calculated from topology diagrams alone, since atomic coordinates are required to compute it. To quantify the locality of the topology from the topology diagram alone, we introduce a variant of RCO, the *β*-sheet-topology Relative Contact Order (*β*RCO). It is defined as

$$\begin{array}{c} \beta RCO=\frac{1}{N_{\beta }\cdot N_{\text{pair}}}\sum^{N_{\text{pair}}}\Delta M_{i,j}, \end{array}$$

where *N*_*pair*_ is the total number of hydrogen-bonded *β*-strand pairs, Δ*M*_*i*,*j*_ is the sequence separation between hydrogen-bonded *β*-strands *i* and *j*, and *N*_*β*_ is the total number of *β*-strands in the protein topolgy.

### 4.5 The lattce HP model and its variant with local interactions

Both the sequence and conformational space of proteins in nature are so large that even today it is impossible to explore them all. To understand the essense of sequence-structure relationships of proteins, several coarse-grained models of proteins have been developed. Even though these models offer a coarse-grained depiction, they yield valuable insights into various aspects of natural proteins [42, 43]. Among them, the lattice HP model [29] has played an important role for understanding sequence-structure relationships [31].

In the lattice HP model, protein chains are configured as self-avoiding walks on two-dimensional square lattices. Based on the assumption that the hydrophobic interaction is the dominant force in protein folding [44], the model considers only two types of amino acids: hydrophobic (H) and polar (P). A protein is represented as a specific sequence of H and P monomers (for example, HHHHPHPPHPHPPHHH). This simplistic representation enables us to conduct a comprehensive exploration of the interplay between protein sequences and structures, particularly for short protein chains. Each interaction between two H monomers that are adjacent in space but not covalently linked is favored by a constant energy −*ϵ*_HH_, and all other interaction energies are zero. Here, *ϵ*_HH_ is a positive constant. The total energy, denoted as *E*_HP_(Γ), associated with a protein chain’s conformation Γ is solely determined by the number of hydrophobic–hydrophobic contacts, represented as *n*_HH_(Γ). This energy is expressed as

$$\begin{array}{c} E_{\text{HP}}\left(\Gamma \right)=-\epsilon_{\text{HH}}\cdot n_{\text{HH}}\left(\Gamma \right). \end{array}$$

Althogh the HP model accounts for hydrophobic interactions, the dominant force in protein folding, it ignores the conformational rules as shown in Fig 2A–2C. To elucidate the impact of these rules on the sequence-structure relationships, we introduce a protein model that integrates additional interactions into the original HP model to mimic the rules illustrated in Fig 2A–2C. We define three specific local structures shown in Fig 4A–4C, as inherently unfavorable structures. The reasons for choosing these three structures as unfavorable structures are discussed in the next section. Each local structure is subject to a positive energy penalty, denoted by *ϵ*_penalty_, irrespective of its amino acid sequence. The total energy penalty for a given structure Γ is given by

$$\begin{array}{c} E_{\text{penalty}}\left(\Gamma \right)=\epsilon_{\text{penalty}}\cdot n_{\text{penalty}}\left(\Gamma \right), \end{array}$$

where *n*_penalty_(Γ) denotes the number of such unfavorable structures within a given configuration Γ. Note that rotating within the plane or horizontally or vertically flipping the three conformations incurs penalties in the same way. Accordingly, the total energy of the modified HP model, *E*(Γ), corresponding to a given protein structure Γ, is determined by the relationship

$$\begin{array}{c} E\left(\Gamma \right)=E_{\text{HP}}\left(\Gamma \right)+E_{\text{penalty}}\left(\Gamma \right). \end{array}$$ (1)

S17A Fig shows an example of the amino acid sequence of this model and S17B, S17C, S17D and S17E Fig present its structure with (*n*_HH_, *n*_penalty_) values of (0,1), (4,2), (8,1), and (8,0), respectively.

In this investigation, protein chains comprising 16 monomers were analyzed, resulting in a total of 802,075 conformations for this chain length. The selection of 16 monomers as the chain length was based on the critical role of the surface-to-interior ratio in conformation for protein folding [45]. An ideal ratio is approximately 1/4 for a typical globular protein [45]. In a two-dimensional framework, a chain of 16 monomers can fold into a conformation where 25% of the monomers are located in the interior core (four interior sites within a 4 × 4 maximally compact structure). Consequently, the two-dimensional chains examined herein aptly represent the appropriate ratio of internal to external monomers.

If we set the chain length to 16, the total number of possible amino acid sequences in the model reaches 2^16^. For each of these 2^16^ sequences, we calculated the energies of all 802,075 possible structures using Eq (1) to find protein-like sequences. Here, a ‘protein-like sequence’ is a sequence that has only one lowest energy conformation for a given sequence. This criterion is based on the characteristic of globular proteins, which have only one conformation known as the native structure, possessing the lowest free energy.

### 4.6 The relationship between the three unfavourable structures of the lattice model and the rules derived from the database analysis

This section elucidates the relationship between the three unfavourable structures of the lattice model shown in Fig 4A–4C and the rules derived from the database analysis.

Initially, we will briefly summarize the findings of the database analysis. The three substructures illustrated in S18A Fig—a left-handed *βαβ* motif, an antiparallel *αβ*-unit, and a negative register shift—are less common in the dataset (about 2%, 35%, and 5%, respectively) and have a lower likelihood of being generated in simulations, as discussed in the results section. These observations imply that the three substructures reside in a high free energy state. However, it is important to note that these three substructures do not possess a sufficiently high free energy to be completely prohibited. In the results section, we demonstrated that an F*αβ*P (S18B Fig) is frustrated, i.e., it necessarily contains one of the three structures. For instance, S18C Fig illustrates that Rule II is violated (an antiparallel *αβ*-unit is included) in an F*αβ*P, regardless of the orientation of the side chains of the N-terminal residues of the two strands, when the height of the N-terminal residue of the two *β*-strands is the same.

Subsequently, we introduce two rules for demonstrating that the reverse structure of an F*αβ*P (S18D Fig) is not frustrated. The rules needed here are the reversed chain direction variants of Rule II and Rule III. Rule I is omitted below since the same structure is obtained by reversing the chain direction of a *βαβ*-unit. The first rule is the reversed chain direction variant of Rule II, which addresses the orientation of *βα*-units. S19A Fig shows occurrence frequencies of antiparallel or parallel orientation of *βα*-units in the dataset. Here, the orientation of a *βα*-unit is defined as parallel if the vector from the *β*-strand to the *α*-helix of a *βα*-unit aligns parallel to the C*α*-C*β* vector of the last residue in the *β*-strand. Conversely, when the two vectors are antiparallel, the orientation of a *βα*-unit is defined as antiparallel. A noteworthy aspect of this histogram is that both parallel and antiparallel orientations occur at approximately the same frequency in *βα*-units. This pattern was also reported in simulations (please refer to Ref. [23]), suggesting that both antiparallel and parallel orientations are physically equally probable in *βα*-units. Based on these observations, it is reasonable to assume that the rule for *βα*-units is equally probable for both antiparallel and parallel orientations. We refer to this rule as Rule IV (the *βα*-rule). The second rule is the reversed chain direction variant of Rule III, which addresses the C-terminal register shift between the *β*-strand of a *βα*-unit and its neighboring *β*-strand. S19B Fig shows occurrence frequencies of negative, zero, and positive register shifts of C-terminal register shift for the *β*-strand of an *βα*-unit and its neighboring *β*-strand. Here, C-terminal register shift is defind as the residue offset between the C-terminal residue of the *β*-strand of the *βα*-unit and the C-terminal residue of the *β*-strand to its right when viewed from a specific direction. This specific direction is defined as the one where the *α*-helix of the *βα*-unit is closer to the *β*-strand and the *β*-strand is facing upwards (S19B Fig). The sign of the register shift is defined as negative when the C-terminal residue of the *β*-strand of the *βα*-unit is located above the C-terminal residue of the *β*-strand to its right, and positive when the opposite is the case. The histogram of C-terminal register shift resembles that of N-terminal register shift depicted in Fig 2: The most frequent occurrence is the zero shift, while a negative shift is rare. Currently, it is unclear whether this behaviour arises from physical interactions or other influences. Nevertheless, given the frequent occurrence of C-terminal register shift zero, it seems reasonable to conclude that shift zero is physically favored. We refer to this rule as Rule V (the C-termial register shift rule).

Now, in light of these two new rules, we demonstrate that the reverse structure of an F*αβ*P (S18D Fig) is not frustrated. To confirm that this structure is not frustrated, it suffices to demonstrate that there exists a configuration that satisfies both rules simultaneously. Since it is assumed that the right-handed rule (Rule I) is always followed, this rule is not explicitly mentioned below. For instance, consider the case where the C-terminal residues of the two *β*-strands forming the parallel *β*-sheet have the same height, resulting in a zero register shift (see S18E Fig). This zero register shift ensures that Rule V is satisfied. Furthermore, it is evident that Rule IV is also fulfilled, as both parallel and antiparallel orientations are physically favored in *βα*-units. As the configurations shown in S18E Fig satisfy both rules simultaneously, we concluded that the reverse structure of an F*αβ*P (S18D Fig) is not frustrated.

Based on the previous explanations, let us discuss the relationship between the three structures depicted in Fig 4A–4C, chosen as unfavourable in the lattice model, and the rules obtained in the database analysis. Database analysis has revealed three facts: (i) the three substructures (depicted in S18A Fig) are in a high free-energy state; (ii) an F*αβ*P invariably contains one of the three substructures, i.e., it is frustrated; (iii) the reverse structure of an F*αβ*P is not frustrated. To replicate these three properties, a variant of the lattice HP model should be constructed as follows: (a) in accordance with fact (i), the three substructures should be selected as energetically high states; (b) in accordance with fact (ii), there must be a frustrated substructure; (c) in accordance with fact (iii), the reverse structure of the frustrated substructure must not be frustrated. The three local structures depicted in S18F Fig were selected as one of the simplest sets of substructures that meet these requirements. Indeed, an FLS (S18G Fig) is frustrated because all possible configurations resulting from adding one residue to the C-terminal side of an FLS result in three unfavorable structures (see S18H Fig). Therefore, unless this substructure is not at the C-terminus of the entire chain, it is frustrated. Furthermore, a reverse FLS (depicted in S18I Fig) is not frustrated. The structure depicted in S18J Fig represents a configuration where one residue has been added to both the N- and C-terminal sides of a reverse FLS. This structure does not contain the three unfavorable structures. Therefore, a structure containing a reverse FLS can be devoid of the three unfavorable structures, proving that a reverse FLS is not frustrated. Taken together, an FLS and an F*αβ*P are analogous in that both are frustrated, while their reverse structures are not frustrated.

## Supporting information

S1 Fig Occurring Frequency of Homologous-group in a Topology. (A) OFHT for topologies with N_F*αβ*P_ = 0. (B) OFHT for topologies with N_F*αβ*P_ > 0. (C) OFHT for topologies with *N*_*j*_ ≤ 1. (D) OFHT for topologies with *N*_*j*_ ≥ 1.
(EPS)

S2 Fig Topology Diagrams of Various States. Diagrams of the state with (N_F*αβ*P_, *N*_*j*_) = (0,3), those with (N_F*αβ*P_, *N*_*j*_) = (0,4), and those with (N_F*αβ*P_, *N*_*j*_) = (0,5).
(EPS)

S3 Fig Topology Diagrams of a Specific State. Diagram of the state with (N_F*αβ*P_, *N*_*j*_) = (1,2).
(EPS)

S4 Fig Topology Diagrams of a Specific State. Diagram of the state with (N_F*αβ*P_, *N*_*j*_) = (1,3).
(EPS)

S5 Fig Topology Diagrams of a Specific State. Diagram of the state with (N_F*αβ*P_, *N*_*j*_) = (1,4).
(EPS)

S6 Fig Topology Diagrams of a Specific State. Diagram of the state with (N_F*αβ*P_, *N*_*j*_) = (1,5).
(EPS)

S7 Fig Topology Diagrams of Various States. Diagrams of the state with (N_F*αβ*P_, *N*_*j*_) = (2, 2), those with (N_F*αβ*P_, *N*_*j*_) = (2, 3), those with (N_F*αβ*P_, *N*_*j*_) = (2, 4), and those with (N_F*αβ*P_, *N*_*j*_) = (2, 5).
(EPS)

S8 Fig Effect of *βRCO* value and presence of long-distance crossover connection on OFHT. (A) The scatter plot of the *βRCO* and OFHT for all the 167 clash-free topologies. Topology diagrams of frustration-free topologies are shown around the graph. The numbers below the topology diagrams represent their *βRCO*s. long-distance crossover connections are depicted by magenta curves. The data for each frustration-free topology is indicated by a gray arrow. (B) Classification of frustration-free topologies. All the 14 frustration-free topologies are classified according to whether the *βRCO* value was greater or less than 0.2 and whether they contain a long-distance crossover connection or not. The numbers below the topology diagrams represent their OFHTs. Topology diagrams surrounded by a red box represent superfolds.
(EPS)

S9 Fig Histogram of Designability for the Lattice Model with Local Interactions. (A) Histogram of designability for structures with *N*_FLS_ = 0. (B) Histogram of designability for structures with *N*_FLS_ > 0. (C) Histogram of designability for structures with *N*_c_ ≥ 7. (D) Histogram of designability for structures with *N*_c_ ≤ 6.
(EPS)

S10 Fig The relationshitp between the relative contact order and designability for all the self-avoiding 802,075 conformations of the lattice model. The parameters *ϵ*_HH_ = 1.0 and *ϵ*_penalty_ = 2.0 were used for energy calculation.
(EPS)

S11 Fig Relationship between designability and various quantities derived from the lattice model with the parameters *ϵ*_HH_ = 1.0 and *ϵ*_penalty_ = 0.1. (A) Shows a histogram of designability for the lattice model. (B) Designabilities of structure pairs are presented, where one is a structure that does not contain an FLS and the other is its reverse topology that does. (C) Histogram of designability for structures with *N*_FLS_ = 0. (D) Histogram of designability for structures with *N*_FLS_ > 0. (E) Histogram of designability for structures with *N*_c_ ≥ 7. (F) Histogram of designability for structures with *N*_c_ ≤ 6. (G) Illustrates the number of highly designable structures (*N*_*s*_ > 100) mapped onto the two-dimensional grid, *N*_F*αβ*P_ and *N*_FLS_. (H) The scatter plot of the RCO and designability for all the self-avoiding 802,075 conformations.
(EPS)

S12 Fig Relationship between designability and various quantities derived from the lattice model with the parameters *ϵ*_HH_ = 1.0 and *ϵ*_penalty_ = 0.5. (A)-(H) are the same as in S11 Fig, except for the values of the parameters.
(EPS)

S13 Fig Relationship between designability and various quantities derived from the lattice model with the parameters *ϵ*_HH_ = 1.0 and *ϵ*_penalty_ = 1.0. (A)-(H) are the same as in S11 Fig, except for the values of the parameters.
(EPS)

S14 Fig Relationship between designability and various quantities derived from the lattice model with the parameters *ϵ*_HH_ = 1.0 and *ϵ*_penalty_ = 3.0. (A)-(H) are the same as in S11 Fig, except for the values of the parameters.
(EPS)

S15 Fig Relationship between designability and various quantities derived from the lattice model with the parameters *ϵ*_HH_ = 1.0 and *ϵ*_penalty_ = 4.0. (A)-(H) are the same as in S11 Fig, except for the values of the parameters.
(EPS)

S16 Fig An example of the structure violating the Rule II. (A) Displayed here is the 2-176 residues of chain A of adenylate kinase from Methanococcus igneus (PDB ID: 6PK5). Rainbow coloring from blue to red indicates the N- to C-terminal position of the *β*-sheet region. C*β* atoms of the *β*-sheet region are depicted as spheres. (B) Schematic representation of the stucture of the adenylate kinase from Methanococcus igneus. The arrows represent *β*-strands and the rectangles represent *α*-helices. The square with a circle inside symbolizes a single amino acid residue with a side chain located on the proximal side, and the colored filled square represents a residue with a side chain located on the opposite side. The region violating the Rule II is outlined in magenta.
(EPS)

S17 Fig An example of the modified HP protein model. (A) displays an example of the amino acid sequence of the model. (B)-(E) show the examples conformation of the HP sequence indicated in (A). The filled red and blue circles represent H (hydrophobic) and P (polar) residues, respectively. The N- and C-terminal residues are labeled ‘N’ and ‘C’. Black lines with arrows depict covalent bonds; the arrows point from the N- to the C-terminal end of the chain. Green dotted lines illustrate hydrophobic–hydrophobic contacts, and gray areas highlight physically unfavorable local regions defined in Fig 4. (B), (C), (D), and (E) present structures with (*n*_HH_, *n*_penalty_) values of (0,1), (4,2), (8,1), and (8,0), respectively.
(EPS)

S18 Fig The relationship between the three unfavorable substructures of the lattice model and the rules derived from the database analysis. (A) Physically unfavorable structures identified by database analysis. The arrows represent *β*-strands and the rectangles represent *α*-helices. The square with a circle inside represents a single amino acid residue with a side chain located on the proximal side, and the colored filled square represents a residue with a side chain located on the opposite side. (B) A schematic representation of an F*αβ*P. (C) Schematics of structures of an F*αβ*P. In the respective figures on the left and right, Rule II is violated by an *αβ*-unit; the former consists of a green *α*-helix and an orange *β*-strand, while the latter comprises a blue *α*-helix and a cyan *β*-strand. The regions breaking Rule II are outlined in magenta. (D) A schematic representation of a reverse F*αβ*P. (E) Schematics of structures of a reverse F*αβ*P. (F) The physically unfavorable local structures that we defined in the lattice model. Each filled circle symbolizes a single residue. Black lines with arrows depict covalent bonds; the arrows indicate the the N- to the C-terminal chain direction. Rainbow coloring from blue to red indicates the N- to C-terminal position of the residues in the model. (G) A structure of an FLS. (H) All possible configurations resulting from the addition of one residue to the C-terminal side of an FLS. Gray-shaded areas denote FLSs, whereas magenta-outlined regions delineate the physically unfavorable local structures. (I) A structure of a reverse FLS. (J) An example of a configuration in which one residue has been appended to both the N- and C-terminal sides of a reverse FLS. Gray-shaded areas denote a reverse FLS.
(EPS)

S19 Fig Occurrence frequencies of the orientation of *βα*-units and C-terminal register shifts in the dataset. (A) Occurrence frequencies of antiparallel and parallel *βα*-units in the dataset. Displayed below the histogram are schematic diagrams of the antiparallel and parallel *βα*-units. The arrows represent *β*-strands and the rectangles represent *α*-helices. The square with a circle inside represents a single amino acid residue with a side chain located on the proximal side, and the blue-colored filled square represents a residue with a side chain located on the opposite side. (B) Occurrence frequencies of C-terminal register shifts between a *β*-strand of an *βα*-unit and its neighboring *β*-strand in the dataset. Dipicted below the histogram are a schematic representation of negative, zero, and positive C-terminal register shifts.
(EPS)
